# DNA fingerprinting at farm level maps rice biodiversity across Bangladesh and reveals regional varietal preferences

**DOI:** 10.1038/s41598-018-33080-z

**Published:** 2018-10-08

**Authors:** Tobias Kretzschmar, Edwige Gaby Nkouaya Mbanjo, Grace Angelique Magalit, Maria Stefanie Dwiyanti, Muhammad Ashraful Habib, Maria Genaleen Diaz, Jose Hernandez, Zenaida Huelgas, Maria Luz Malabayabas, Subrata Kumar Das, Takashi Yamano

**Affiliations:** 1 International Rice Research Institute (IRRI), Pili Drive, Los Baños, 4031 Laguna Philippines; 2 Present Address: Southern Cross University, Military Road, East Limore, 2480 NSW Australia; 3 Present Address: Graduate School of Agriculture, Hokkaido University, Sapporo, Hokkaido 060-8589 Japan; 4 IRRI Bangladesh Office, 103, Block-F, Rd No 1, Dhaka, 1213 Bangladesh; 5 Institute of Crop Science, College of Agriculture and Food Science, UPLB, College, Laguna, Philippines; 6 Present Address: Asian Development Bank (ADB), 6 ADB Ave, Ortigas Center, Mandaluyong, 1550 Metro, Manila Philippines

## Abstract

The development, dissemination, and adoption of improved rice varieties are imperative for global food and nutritional security. Knowledge of the crop’s distribution across agro-ecologies is important for impact assessment studies, varietal replacement strategies, and the development and implementation of agricultural policies. Bangladesh is the world’s 4^th^ largest rice producer. Though traditional varieties (TVs) are abundant and valued throughout Bangladesh, population growth and vulnerability to climate change, necessitate efficient deployment of high-yielding stress-tolerant modern varieties (MVs). To aid agricultural policy and strategy this study aimed to accurately assess the distribution of MVs and TVs across Bangladesh during the rainfed rice-growing season. Information derived from a survey of rice production areas were compared and combined with DNA fingerprinting information from the same locations. Biodiversity of Bangladesh rice remained high. While TVs and first generation MVs of Bangladeshi and Indian origin were still commonly grown, recently released stress-tolerant MVs were adopted in large proportions in several districts. Although farmers successfully distinguished TVs from MVs grown in their fields, a considerable lack of authenticity among MVs was observed, pinpointing shortcomings in the seed supply chain. This study identifies focal points for extension work and validates DNA fingerprinting as reliable method for impact assessment studies.

## Introduction

The continuous growth of the world population requires a constant increase in rice production to meet human needs. Rice is the primary staple food, particularly for the poor in South Asia and Southeast Asia^1,2^. With more than 30 million metric tons of production in 2014, Bangladesh is the fourth largest rice producer in the world and rice consumption accounts for more than 60% of the average caloric intake of the population^3,4^.

Bangladesh has a land surface of 148,460 km^2^ and more than 161 million inhabitants. The current population is expected to reach 238 million by 2050^3,5^. The country consists of eight divisions: Barisal, Chittagong, Dhaka, Khulna, Mymensingh, Rajshahi, Sylhet, and Rangpur, all of which grow rice. Bangladesh is bordered by India, Burma (Myanmar), and the Bay of Bengal. The country has three agricultural seasons: aman, boro, and aus. Aman is the main rainy season when rice farmers grow both traditional and modern varieties of rice. In the boro season, farmers rely on irrigation water, mostly from tube wells, and grow short-duration modern rice varieties such as BRRI dhan28. Aus is a pre-aman season when upland rice is grown under rainfed conditions.

Food self-sufficiency and food security are the main pillars of the country’s food policy, and rice production has been given high priority. In recent years, Bangladesh has been self-sufficient for rice, but this is threatened by global warming and the increasing population pressure. Bangladesh is dominated by the fertile Ganges-Brahmaputra delta and its proximity to the Himalayas makes it prone to recurrent flooding damage from monsoonal rains, tropical cyclones, snow and glacier melt, upstream water flow, and tidal surges^6^. Floods lead to crop losses of up to four million tons per year^7^. Environmental changes associated with global warming are expected to significantly aggravate this situation^8^. Therefore, the development and adoption of high-yielding modern varieties (MVs) that are flood-tolerant are needed.

Since its independence in 1971, approximately 100 MVs have been officially developed, 78 BR and BRRI dhan varieties released through the Bangladesh Rice Research Institute (BRRI) and 17 Bina dhan varieties released through the Bangladesh Institute of Nuclear Agriculture (BINA) (BRRI personal communication). Approximately 30 of those were specifically developed for the aman season^5^. Breeding and variety release have largely been a public effort under the Bangladesh seed production policy, while imports and distribution of inbred rice through the private sector were limited. Under the New Agricultural Extension Policy (NAEP) in 1996, a centralized extension system was replaced with a decentralized system allowing NGOs and private agribusinesses to contribute to extension delivery service. As opposed to traditional Bangladeshi landraces, post-green revolution MVs are short in height, short in duration, high yielding, and input intensive, albeit less adapted to specific local environments and consumer preferences.

Recent advancements in molecular breeding techniques have facilitated the development of varieties with desirable traits^9,10^. As a result, an increasing number of rice varieties have been released to farmers, sometimes being conversions of older varieties that have been improved via introgression of a single trait. Submergence-tolerant rice varieties are typical examples. They possess a single major quantitative trait locus (QTL) responsible for submergence tolerance, named *Sub1*^11,12^. In the early 2000s, rice scientists successfully introgressed *Sub1* into a popular Indian rice variety called Swarna through marker-assisted backcrossing (MAB)^11,13^. Since then, *Sub1* has been introgressed into several popular rice varieties as it apparently had no negative trade-offs in agronomic performance, grain yield, and grain quality under non-stress conditions but fortified the variety against prolonged submergence^11^. Two Sub1 varieties, BRRI dhan51 (Swarna_Sub1) and BRRI dhan52 (BR11_Sub1), were released in Bangladesh in 2011. Their successful adoption was expected to increase the productivity and income of farmers as previously observed for Sub1 varieties released in India^12^. However, to date, no comprehensive studies on adoption rates for Swarna_Sub1 and BR11_Sub1 for Bangladesh have been undertaken.

Assessing the distribution of existing varieties and monitoring the adoption of new varieties not only contribute to our understanding of the impact of crop improvement in food security and income growth, but also aid research prioritization and inform variety replacement strategies. Impact assessment is typically done by estimating the total area and total number of users of a certain variety and has traditionally relied mainly on breeders’ and farmers’ morphological descriptors and naming. Those, however, have been proven to provide inconsistent results, since morphological descriptors are affected by environmental conditions and naming is error-prone, particularly in informal seed systems. Without a formal seed naming system, farmer identification can vary, which will eventually lead to inconsistencies^14^. The situation is even more complicated in countries with open borders, such as India, Bangladesh, and Nepal, insofar as rice varieties developed in one country could move across borders without going through a proper release system. Therefore, clear distinctions between varieties grown are needed to assess the diffusion of improved varieties^15,16^. These challenges can be overcome through the use of molecular markers.

Molecular markers are useful for precise, rapid detection and exploitation of DNA polymorphism. Molecular genetic techniques have evolved from low-throughput to high-throughput marker platforms. A range of molecular marker systems have been used for fingerprinting and characterizing varieties and germplasm accessions, including but not limited to random amplified polymorphic DNA (RAPD)^17^, amplified fragment length polymorphism (AFLP)^18^, inter-simple sequence repeats (ISSR), simple sequence repeats (SSR)^19,20^, SNP and diversity arrays technology (DArT)^21^. Next-generation sequencing, high-quality reference genome of cultivated *indica* and *japonica* rice combined with genomic resequencing data for thousands of accessions have provided extensive single nucleotide polymorphism pools to select informative markers for different sets of germplasm^22–24^. Unlike SSRs, single base-pair substitutions occur in the genome at higher frequency and are bi-allelic. The development of highly efficient, robust, and cost-effective multiplexed fixed array platforms such as the Illumina Infinium rice 6K chip^25,26^ has further propelled the adoption of SNPs as the marker system of choice^27–29^.

Here, we employed the Illumina Infinium rice 6K chip (C6AIR)^25^ to assess the distribution of Bangladeshi MVs (BMVs) across small and medium farms in six divisions of the country, with a particular focus on submergence-tolerant lines. The study highlights the importance of DNA fingerprinting for reliable and accurate assessment of varietal distribution. We report substantial discrepancies between varieties reported to be grown by farmers and what they were growing. That is the variety name a farmer gave to the variety he was growing did often not match the identity of the sample according to DNA fingerprinting. We show that older generation modern varieties and traditional varieties are still widely used despite the release of several new improved varieties.

## Results

### Sample collection, plant materials, and quality control procedure

A total of 24 breeder seeds and 1,282 farmers’ samples were collected and genotyped using the Infinium 6K. The breeder seed collection contained 18 aman varieties and 6 boro/aus varieties popular during aman season. They were officially released through either BRRI or BINA (Supplementary spreadsheet A) as early as 1980 (BR10 and BR11) and as recently as 2011 (BR57). In particular, it contained BRRI dhan51 and BRRI dhan52, both released in 2010, which contain the Sub1 gene and are referred to as Swarna_Sub1 and BR11_Sub1.

Quality control criteria (He >5%, call rate >0.8) implemented on genotype data pinpointed 107 samples with heterozygosity higher than 5% and 6 samples with genotype call lower than 0.8, which were discarded from downstream analyses. A total of 1,171 farmers’ samples and 24 breeder seeds were retained for further analyses (Supplementary spreadsheet B). Of the 4,606 SNPs on the 6K chip, 4,503 SNPs with a minor allele frequency greater than 0.01 (MAF >0.01) were used for subsequent analyses.

**Figure 1:**
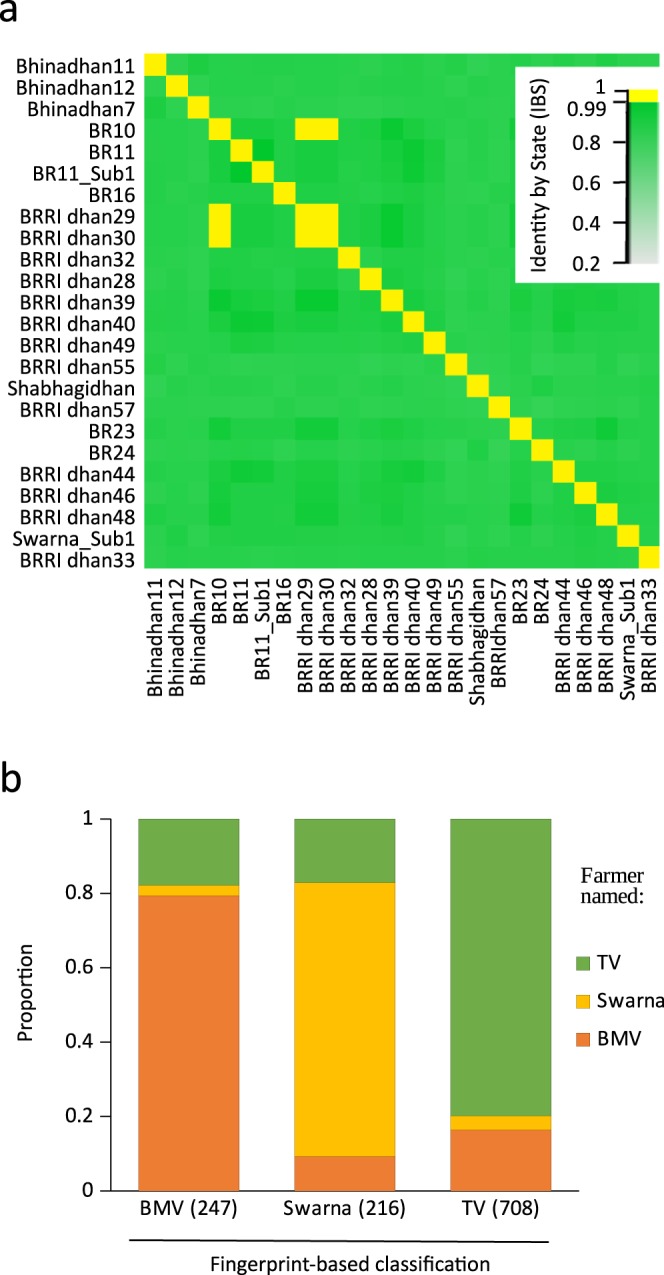
Basic characterization of breeders’ seed and aman samples by fingerprinting (**a**) Heat map of identity-by-state (IBS) of 24 breeders’ seed reference samples. Yellow depicts identity of 0.99–1.00, dark green depicts high relatedness, and light green depicts low relatedness. (**b**) Classification of aman samples by fingerprinting into Bangladeshi modern varieties (BMVs (247)) having >98% similarity with a breeders’ seed reference; Swarna (216) having <98% similarity with a breeders’ seed reference; and Swarna as the closest match in the 3K reference set; and traditional varieties (TVs (708)) having <98% similarity with a breeders’ seed reference. Proportion of respective farmers’ naming of samples as BMVs (orange), Swarna (yellow), or TVs (green) is shown.

Pairwise comparisons between the 24 breeder seeds revealed identity-by-state (IBS) distances that ranged from 0.8 to 1.0, with an average of 0.9+/−0.03 (Fig. 1a). BRRI dhan29 and BRRI dhan30 were duplicates (IBS = 1.000), and highly similar to BR10 (IBS = 0.999). BR11 and its conversion line BR11_Sub1 displayed an IBS distance of 0.986 (Supplementary spreadsheet C).

### Variety association through farmer information

Farmers reported a total of 213 variety names. More than half of these (118) were associated with only one or two samples (Supplementary spreadsheet D). Of the 1,171 samples retained for analyses, 31.8% (372) were named by farmers as BMVs, as released through BRRI and BINA. The most-represented BMV names were BR11 (5.6%), BR11_Sub1 (4.3%), BRRI dhan49 (3.5%), and Swarna_Sub1 (3.2%). Our breeders’ reference set did not contain 6 of the 27 BMVs reported by farmers (Bina dhan8, BR20, BR22, BRRI dhan34, BRRI dhan41, and BRRI dhan42 (Supplementary spreadsheet D), because they were not available from BRRI or BINA upon request at the time of the study. On the other hand, BRRI dhan57, Sahbhagi dhan, and Bina dhan12, available in the breeders’ reference set were not represented among the farmer names (Supplementary spreadsheet D).

A total of 68.2% of the 1,171 samples had traditional/local names. The most-represented varieties were Guti Swarna (6.8%), a derivative of Swarna; Kalijira (3.8%), referring to a premium, aromatic, short-grain rice; and Pajam (2.7%), likely referring to a high-yielding variety of Malaysian origin developed under an FAO project in the 1960s^30^ (Supplementary spreadsheet D). A total of 16.5% of all the samples contained “Swarna” under different variations (Swarna (24), Guti Swarna (80), Suman Swarna (26), Lal Swarna (17), Babuswarna (11), Sada Swarna (10), Nepali Swarna (7), Panpataswarna (5), Lal Guti Swarna (4), Mamun Swarna (2), Unusswarna (2), Ajgobiswarna (1), Pahari Swarna (1), and Sadhuswarna (2)). Other common names and variations thereof included “Mota” (6.2%), referring to the round bold grain preferred in the western divisions, and “Digha” (2.6%) or “Dighi” (1.4%), referring to tall deepwater rice (Supplementary spreadsheet E).

### Genetic similarity estimate

The comparison of farmers’ samples with the breeders’ seed reference material using DNA fingerprinting analyses showed that 21.1% matched with 15 breeders’ seed references at a similarity of 98% or higher and were considered BMVs (Supplementary spreadsheet F). Of the 78.9% that did not match with the breeders’ seed reference set, 18.4% matched closest with Swarna at an average similarity of 94.6%. The remaining 60.5% were considered TVs (Fig. 1b, Supplementary spreadsheet G and H).

Among BMVs, 79.4% were correctly named by the farmers as BMVs (although not necessarily the correct BMVs), while 17.8% were named as TVs and 2.8% were named as Swarna (Fig. 1b). Similarly, 79.8% of the TVs were correctly named by the farmers as TVs, with 16.4% named as BMVs and 3.8% as Swarna (Fig. 1b). For Swarna, correct naming was slightly lower at 73.6%, with 17.1% named as TVs and 9.3% named as BMVs (Fig. 1b).

**Figure 2:**
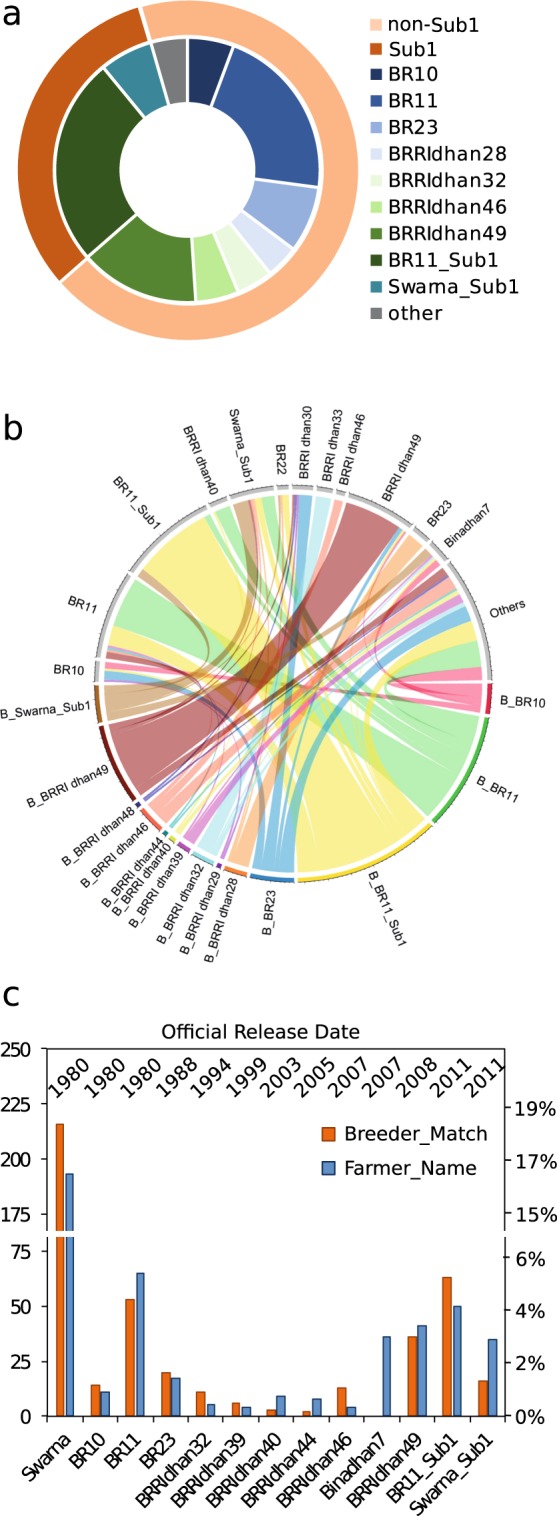
Bangladesh modern variety (BMV) identification through breeders’ seed reference matches (A) and comparison to farmer naming (B and C) (**a**) The 10 most common breeders’ seed matches among 247 BMVs. The outer circle depicts the presence of Sub1 in dark orange and the absence of Sub1 in light orange. (**b**) Sample-by-sample comparison of BMVs by breeders’ seed matches (bottom half) with farmer name (top half). (**c**) Common aman BMVs and Swarna as identified by fingerprinting (orange) and farmer naming (blue). Sample number is displayed on left x-axis and relative proportion on right x-axis. Top shows release date.

Among the identified BMVs, the most popular were BR11_Sub1 (24.7%), BR11 (21.5%), and BRRI dhan49 (14.2%) (Fig. 2a, Supplementary spreadsheet I). Swarna_Sub1 was identified at 6.5%, bringing the total of Sub1 conversions among BMVs to 31.2% (Fig. 2a). The most recent breeders’ seed releases, Sahbhagi dhan and BRRI dhan57, could not be matched with farmers’ samples. The 247 BMV samples were distributed over 46 variety names as per farmer identification, and a considerable amount of the farmers’ samples with different names were genetically identical and vice versa (Fig. 2b; Supplementary spreadsheet I). In more than 50% of the cases in which the farmer was indeed growing a BMV by fingerprint, it was named as a different BMV or, in a few cases, even as a TV (Fig. 2b; Supplementary spreadsheet I). This resulted in an overestimation of certain varieties while others were underestimated. Although more farmers were growing BR11_Sub1 than they reported growing, fewer farmers were growing BR11 and Swarna_Sub1 (Fig. 2c). Although more than 30 farmers were reported to be growing Bina dhan7, not a single match could be made with the respective breeders’ references.

**Figure 3:**
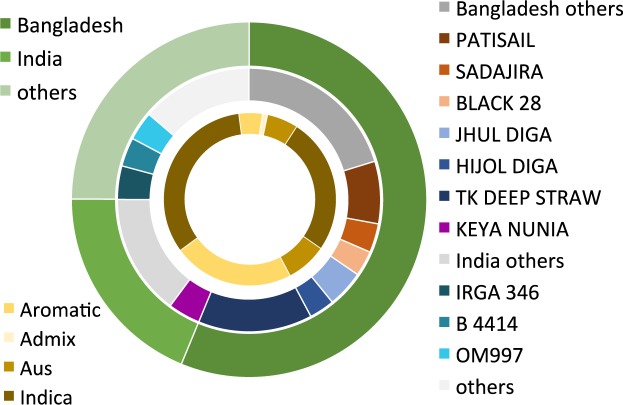
Traditional variety (TV) matching with 3K reference set Closest matches of 708 TV samples with the 3K reference set. Outer circle depicts samples matching with Bangladeshi accessions in dark green, Indian accessions in green, and accessions from other countries in light green. On the inner ring, samples corresponding with the *indica* subgroup are depicted in brown, the *aus* subgroup in light brown, the *aromatic* subgroup in beige, and *admixture* samples in light beige.

With the TV samples that did not match the breeders’ reference set or Swarna, a secondary comparison was performed, using the 3K genomes^23^ as the reference set. A total of 708 samples could be matched to 88 accessions in the 3K set at an average similarity of 93.2%+/− 3.4% (Supplementary spreadsheet J). 3K accessions of Bangladeshi and Indian origin represented 56.2% (398) and 18.8% (133) of the samples (Fig. 3). The ten most common matches covered 361 samples (51% of TVs), six Bangladeshi, one Indian (Keya Nunia), one Colombian (IRGA 346), one Indonesian (B4414), and one Vietnamese (OM997) (Fig. 3). OM997-matching samples were mostly named Bina dhan7 by the farmers, suggesting a relationship. TK Deep Straw, an aromatic landrace, emerged as the Bangladeshi TV in the 3K reference set that matched with the highest number of farmer samples (14.0% of TVs). The majority of the “Mota” samples as well as the Bhushiara samples and many of the Kalijra samples had TK Deep Straw as their closest match, suggesting TK Deep Straw as a “Mota” representative within the 3K set. The second most commonly matched accession was Patisail (7.6% of TVs), and 20 of the 32 samples named by farmers as Pajam closely matched with Patisail, suggesting a relationship. Forty-three of the 45 “Diga”-named samples matched with the 3K *aus* accessions Hijol Diga, Jhul Diga, or Bora Diga of the 3K set, suggesting that in most cases when the farmer referred to a deepwater Digha variety it was indeed Digha.

**Figure 4:**
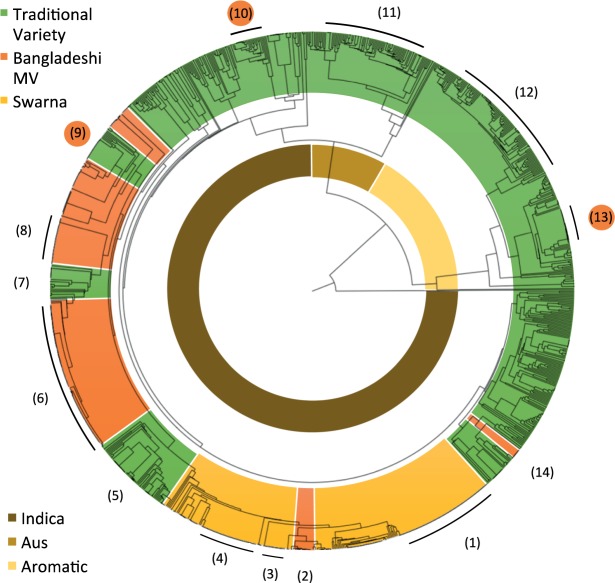
Clustering of Bangladeshi modern varieties (BMVs), Swarna, and traditional varieties (TVs) Unweighted Pair Group Method using Arithmetic Mean-based tree of 1,171 aman samples. On the outer ring, groups of BMVs are depicted in orange, TVs are depicted in green, and Swarna is shown in yellow. Highlighted on the outer ring are clusters of interest by fingerprint match/predominant farmer name: Swarna/Guti Swarna (1), Swarna_Sub1/Swarna_Sub1 (2), Swarna/Babu Swarna (3), Swarna/Suman Swarna (4), Patisail and IRGA 346/Pajam (5), BR11(+/−Sub1)/BR11(+/−Sub1) (6), UPRH265/Horidhan (7), BRRI dhan49/BRRI dhan49 (8), B 4414/BR22 (9), OM997/Bina dhan7 (10), Dhiga/Diga group (11), TK Deep Straw/Mota group (12), KEYA NUNIA/BR34 (13), and BRRI dhan23/BRRI dhan23 (14). Numbers with an orange background indicate BMVs that might have been assigned as TVs due to a lack of breeders’ seed reference. On the inner ring, samples corresponding with the *indica* subgroup are depicted in brown, the *aus* subgroup in light brown, and the *aromatic* subgroup in beige.

Cluster analysis sorted the farmer samples according to their relatedness (Fig. 4). Three *Oryza sativa* subspecies (*aromatic*, *aus*, and *indica*) could clearly be distinguished by the second and third node, respectively. Approximately 75% of the samples were *indica*, including all BMVs. Although more than half of the TVs belonged to the *indica* subspecies (58.3%), a significant proportion were assigned to *aromatic* (27.3%) and *aus* (13.4%) (Figs 3 and 4). BMV samples clustered together, except Swarna_Sub1, which clustered with Swarna and BRRI dhan23, which grouped with TVs. BMVs were interspersed with two clusters of TVs (Fig. 4). One cluster predominantly matched B441 and was commonly named BR22, suggesting that this is indeed a BMV that is lacking a breeders’ seed reference. The other predominantly matched Indian accession UPRH265 and was commonly named Horidhan, a farmer-made variety that became famous in the press and whose origin is still under dispute. Patisail matches clustered together with IRGA 354 matches between Swarna and BMVs (Fig. 4), collectively serving as a proxy for Pajam, the first pre-green revolution Bangladeshi MV. Consistent with being described as round bold and aromatic, most farmer-named Mota and Kalijira accessions clustered with the *aromatic*, which typically share these grain characteristics. Dig(h)a-named samples consistently clustered with the *aus*.

**Figure 5:**
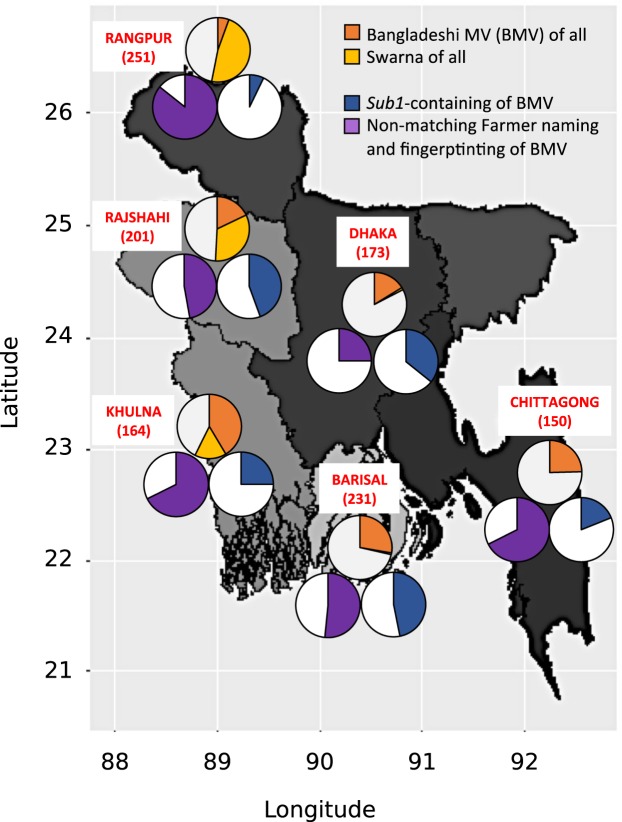
Bangladeshi modern variety (BMV) and Swarna distribution by district Number of samples by district (red). The proportion of BMVs (orange) and Swarna (yellow) is shown in the top circles. Bottom-left circles display the proportion of discrepancy between identification by fingerprinting and farmer naming (purple) among BMVs. Bottom-right circles display the proportion of *Sub1*-containing BMVs (blue) among BMVs. The map was downloaded from DIVA-GIS 7.5 (http://www.diva-gis.org) and modified using ggplot2^43^ in R^44^.

The proportion and type of improved varieties grown by farmers varied considerably between divisions (Fig. 5, Supplementary Table S1). The percentage of BMVs ranged between 5.6% in Rangpur and 41.5% in Khulna, with BR11, BR11_Sub1, or BRRI dhan49 being the most common. Swarna was predominant in Rangpur (47.6%), Rajshahi (32.8%), and Khulna (15.9%), while it was nearly absent in Chittagong, Barisal, and Dhaka. Looking at BMVs and Swarna combined; Khulna had the highest percentage of MVs (57%), while Dhaka had the lowest (17.3%). The percentage of Sub1 varieties among BMVs ranged between 7.1% in Rangpur and 46.9% in Barisal. The BMVs identified by fingerprinting mismatches with farmer identification ranged from 25% in Dhaka to 85.7% in Rangpur.

## Discussion

Correct varietal identification of MVs based on phenotype is difficult, particularly if the varieties are trait conversions (e.g. BR11_Sub1) and relying on farmers’ naming for identification is prone to bias. Both cause distortions in assessments and consequently unreliable predictions and recommendations. Alternatively, farmer elicitation with DNA fingerprinting technology was used to estimate the diversity of TVs and the adoption of MVs, with a particular focus on Sub1-containing materials.

IBS distance analysis confirmed that most breeders’ seed reference samples were unique. BRRI dhan29 and BRRI dhan30, however, were identical, and it remains unclear which sample is true. A single farmer’s sample was named BRRI dhan29, but it matched the BR23 breeders’ seed 100%. Likewise, six out of nine farmer-named BRRI dhan30 samples had a 100% match with BR23, while one matched the BRRI dhan29/BRRI dhan30 breeders’ seed (Supplementary sheet F).

For the 2014 and 2015 survey for the aman season, farmers gave a total of 213 variety names. Although this might seem high, it was considerably less than the 505 varieties reported for the aman season in a 2005 rice biodiversity study^30^. Although this discrepancy could in part be attributed to the significantly smaller sample size in the current study, it nevertheless supported previous observations that the rice biodiversity of Bangladesh is declining^30^. Including Swarna, approximately 50% of the samples were named by farmers as being MVs (Supplementary spreadsheet D and E), which was more than the 45% reported in 2005^30^. Based on farmer identification, this might indicate a shift toward the use of modern varieties during the aman season, which is accompanied by a decline in biodiversity over the last decade. Fingerprinting results, however, suggest that the actual percentage of Swarna and BMVs combined might be as low as 41% (Figs 1b and 4, Supplementary spreadsheet F and G).

Swarna, a variety with Indian origin introduced to Bangladesh through informal cross-border exchange^31^ was most abundant, with 16.4% by naming and 18.4% by fingerprinting (Fig. 2b). Contrastingly, the Sub1 conversion, Swarna_Sub1, was identified at 3.2% by naming and at only 1.4% by fingerprinting (Fig. 2b). BR11_Sub1 and BR11, on the other hand, contributed almost equally to a total of approximately 10% in both the farmer-naming and fingerprinting data set (Fig. 2b). This contradicts the findings of the 2005 report^30^, in which BR11 was most abundant with 26.5% and Swarna accounted for only 11.6%. Collectively, this suggested (i) an underestimation of Swarna, probably then and now; (ii) a continued expansion of Swarna between 2005 and 2015; (iii) replacement of BR11 with recent MVs such as BR11_Sub1 and BRRI dhan49; and (iv) a lack of replacement of Swarna by its conversion line Swarna_Sub1.

Approximately 21% of the analyzed samples matched the breeders’ reference seeds at >98% and hence were considered BMVs. This finding might be biased to some degree, since some farmer-named BMVs might not have been correctly identified as BMVs because of the lack of the respective reference. A total of 46 samples or 4% were named by farmers as BMV for which we did not have reference samples (BRRI dhan34, BRRI dhan41, BRRI dhan42, BR22, Binadhan8) (Supplementary spreadsheet D). BRRI dhan34 named samples largely matched with KEYA NUNIA a common 3K reference match (Fig. 3). For BR22, 9 out of 23 named samples did indeed cluster with BMVs (Fig. 4). For BRRI dhan34, one of the few *aromatic* BMVs, 7 out of 10 farmer-named samples clustered within the *aromatic* subgroup of TVs. Further, it cannot be fully ruled out that incorrectly assigned breeders’ reference samples could have created distortions. This could have been the case for Binadhan7, which, although it was commonly named by farmers, no breeders’ match could be found (Fig. 1a). Instead, 17 out of 36 Binadhan7-named samples matched best with OM997 and clustered within the TVs (Fig. 4). Taking clustering and consistent naming into account could thus bring the percentage of BMVs to around 25%.

The most-represented BMVs were BR11 (4.5%) and BR11_Sub1 (5.2%) (Fig. 2a,c). Introduced in Bangladesh three decades ago, BR11 is the first generation of high-yielding rice (Supplementary spreadsheet A). Although BR11 does not perform well under unfavorable ecosystems such as those having conditions with soil salinity or submergence, it meets overall farmer, miller, and consumer demand and thus has maintained high popularity in the aman season. This could explain the success of BR11_Sub1 over other stress-resilient BMVs. Being nearly identical to its parent BR11 in terms of yield and grain quality; it has the added advantage of survival under full submergence for up to 14 days^32,33^. This does not, however, explain why Swarna_Sub1 has not been adopted well, despite the finding that Swarna was more popular than BR11. It can be speculated that (i) BR11_Sub1 noticeably outperforms Swarna_Sub1, (ii) BR11_Sub1 is easier to obtain than Swarna_Sub1 through official channels, and (iii) Swarna_Sub1 has a penalty over Swarna under non-stress conditions. An interesting speculation regarding (iii) is that Swarna_Sub1 is not adopted well because of background effects. If the local Swarna lines, which differ by up to 8% genetically from the original Indian Swarna, were indeed locally adapted versions of the original, then Sub1 conversions of the original Indian Swarna would be devoid of these local adaptations and hence the resulting Swarna_Sub1 would be inferior to the local Swarna lines. In India, Swarna_Sub1 yield under non-stress conditions was slightly less than for Swarna^12^.

A total of 18.4% of the farmers’ samples had Swarna as the closest match in the 3K set, with an average similarity of 94.6% (Supplementary spreadsheet G). Of these, only 6.5% matched at a similarity of 98% or higher. All others matched at a similarity between 90% and 97%. Distinct lineages of Swarna could be identified by name (Fig. 4 and Supplementary spreadsheet E) and clustering (Fig. 4), and specific Swarna derivative names were predominantly found in a cluster of genetically identical samples (Fig. 4). This suggested that, over the 35 years of its introduction to Bangladesh, Swarna had significantly diversified from its original genotype and that several distinct lineages are continuously propagated. This was not the case for BR11, which retained an average similarity above 99% to the breeders’ reference and high collinearity (Supplementary spreadsheet F). The discrepancy might be explained by the fact that, although certified seed is commonly available for BR11, Swarna seed is exclusively multiplied and distributed through unofficial channels. Farmers usually select the best plants from their field to produce seed and there is a high chance that unintentional outcrossing events have been maintained and amplified through positive farmer selection.

Of the 708 TV samples, more than half (56.4%) matched best with 3K accessions of Bangladeshi origin, some of which served as good proxies for the farmer samples. A total of 18.8% matched Indian accessions (Fig. 3, Supplementary spreadsheet H) and it can be assumed that a proportion of these are indeed landraces of Indian origin. For the most common match, the *aromatic* KEYA NUNIA (Fig. 3), similarities were consistently 97.5% and higher, suggesting near identity with the reference and high collinearity (Fig. 4). This variety was mostly found in Rangpur and Rajshahi, which share borders with West Bengal (India). Interestingly, the most common name given by farmers was BRRI dhan34 (26%). Since BRRI dhan34 was absent from the reference library, mismatching could be possible. Like KEYA NUNIA, BRRI dhan34 is an *aromatic* variety. It is thus likely that Keya Nunia/BR34 was adopted like Swarna through informal cross-border exchange, although the direction of the transfer might have been the opposite. Other common 3,000 genome matches likely represented Bangladeshi varieties BR22 (B 4414), Bina dhan7 (OM997), and Pajam (IRGA 346) (Fig. 4). Even though the 3K set contains 183 Bangladeshi accessions, only 40 of them had close matches, with similarities ranging between 99.7% and 86.1%. Of the common Bangladeshi TVs, only Pajam had a near-perfect match, with similarities with Patisail reaching as high as 99.7% and several Diga varieties matching with Hijol Diga above 98%.

The findings that several farmer-named BMVs were not among the available breeders’ reference set and that the 3K set served as a suboptimal reference set for Bangladeshi TVs highlight the importance of a comprehensive reference library to facilitate sample identification and impact assessment^14^. Combining similarity estimations with phylogenetic analyses (Fig. 4) and common farmer naming helped overcome some of the problems related to the lack of reference materials as MVs tend to have a narrow genetic base and hence cluster together (Fig. 4).

The present study revealed that older generation MVs (varieties released before 1990) and TVs were still widely used by farmers during aman, despite the release and spread of several new improved BMVs^5^ (Fig. 2b). This preference is undoubtedly related in part to their good grain quality, premium prices, satisfactory grain yield, and lodging resistance. On the other hand, this might point to shortcomings in the seed system: (i) the inability of the research system to develop new varieties with traits superior to those of the existing ones that would incentivize farmers and millers to change^30,34,35^, (ii) the low availability of the good quality BMV seeds^34,36^, (iii) the extension system often did not target millers and input dealers^34^, and (iv) the inefficient extension system that fails to get new releases to the farmers in a targeted manner^34^. With more than 15% of the total identified samples, aromatic TVs are widely grown during aman. Some of the traditional cultivars, especially aromatic rice, have extra value for consumers. However, there seems to be a lack of aromatic MVs to replace current traditional cultivars. Newly released MVs should capture this trait value on top of climate resilience to make adoption more likely. For example, BRRI dhan34, a short, bold, and aromatic aman variety, if fortified with Sub1 and properly marketed, might help replace Mota TVs. Traditional rice cultivars, although low yielding, remain valuable genetic resources that could be used for rice improvement to develop products that meet the demand of farmers and consumers alike^5^. Bangladeshi aman TVs remain a good example of *in situ* conservation of rice biodiversity.

Although in more than 80% of the cases farmers were able to distinguish between BMVs and TVs (Fig. 1b), substantial discrepancy was observed among BMVs the farmers reported growing and BMVs they were growing according to genetic fingerprints (Figs 2b,c and 5). Among the samples identified as BMVs, more than 50% were named differently by farmers (Figs 2b and 4), suggesting local assessments based on farmer information to be unreliable. Overall, certain varieties were overestimated and others were underestimated (Fig. 2c). Based on farmers’ identification, the most popular BMV was BR11, whereas, based on fingerprinting, it was BR11_Sub1 (Fig. 2c). Morphologically both varieties are nearly indistinguishable^32,33^. Moreover, based on farmer information only, this study would have concluded that across Bangladesh one-third of the aman season varieties were BMVs, while fingerprinting data suggested that a maximum of one quarter were identified as BMVs. DNA fingerprinting can prevent mis-estimations for impact assessment, provided a proper reference set is available. Similar observations have been reported when assessing the adoption pattern for other crops, including beans^37^, cassava^14^, maize and wheat^38^, as well as sweet potato^39^. Such discrepancies point out the limitations of methods based on farmers’ responses and the necessity to adopt efficient and precise methods for tracking varietal diffusion.

Substantial divisional variation in the choice and the proportion of improved varieties was observed (Fig. 5, Supplementary Table S1), which might inform local varietal replacement strategies. Rangpur displayed the lowest adoption rate of BMVs and nearly half of all samples identified as Swarna. Of the few BMV samples of Rangpur, more than three-quarters did not match farmers’ names and BR11 remained the most popular. Collectively, this would make Rangpur a prime target for the promotion of new-generation BMVs, distributed through official channels. Although Khulna had the highest adoption rate of BMVs, it also had a high proportion of wrong farmer identification. These results might indicate inefficiencies in the distribution and extension system, which could result in an informal spread of recent MVs to local farmers at an increased risk of loss of authenticity. Considering the lack of success of Swarna_Sub1, targeted varietal replacement strategies are needed to counter the predominance of Swarna in Rangpur and Rajshahi. In Barisal, the most common variety match by fingerprinting was TK Deep Straw (28%) (Supplementary Table S1), which was identified as a proxy for Mota varieties. This matches an earlier report^30^ claiming that Mota (a Bengali word for fat) is very popular in the Barisal region because consumers prefer short, bold grains. The most common MV in Barisal was BR11_Sub1, which is also adapted to the agro-ecology of the region, but lacks the consumer-preferred short-grain attribute. Short-grain Sub1 conversions (e.g. BRRI dhan34) could find demand there.

Incorporating modern fingerprinting technology in impact assessment helped to increase the accuracy of assessments in Bangladesh and revealed a considerable lack of authenticity among grown MV. The tendency of farmers to produce their own seed rather than to buy certified stock might explain the lack of authenticity and partially contribute to slow adoption rate of several recent releases. Even though *Sub1*-containing varieties were found in all regions, overall adoption rates were still below 10%. On one hand farmers still tend to prefer traditional varieties that either meet specific consumer demands such as grain shape or aroma (e.g. Mota and Kalijira) or regional stress adaptation. On the other hand, inefficient extension systems reinforced by low availability of recent release MV seeds (e.g. BR11_Sub1) might explain the consistent popularity of older release MV (e.g. BR11) and Swarna. Therefore, improved marketing and/or dissemination strategies for stress-resistant MV varieties need to be more broadly applied in target regions and the benefits of using certified seed of these need to be demonstrated at scale.

The rapid adoption of BR11_Sub1 suggests that agricultural extension policies coupled with improved dissemination technologies are indeed showing effect. In the 6 years since its release, BR11_Sub1 has already surpassed BR11 to become the most popular BMV, and it made up more than a quarter of all BMVs recorded in this study (Fig. 2a,c). The extensive reference provided through this study will undoubtedly help to improve future impact assessment studies, refine agricultural policies, and inform demand-driven varietal replacement strategies.

## Materials and Methods

### Sample collection and DNA extraction

Surveys of rice production areas in Bangladesh were conducted in 2014 and 2015 prior to aman season. The sampled areas were distributed over six divisions across Bangladesh: Barisal, Chittagong, Dhaka, Khulna, Rajshahi, and Rangpur. Survey areas were selected by using agro-ecological zones as the primary stratification and for each agro-ecological zone, households were randomly selected by using district, zone, and village as stratification.

In shorts, in each year 16 rice-producing districts were randomly selected out of the 64 districts in Bangladesh and 75 Thanas (sub-districts controlled by individual police stations) were randomly selected from the 16 districts. In each of the 75 Thanas, one village was randomly selected from the census and in each village, 10 households were randomly selected from the village information and farmers baseline.

Four out of the 10 households from each village were randomly selected for seed collection. Consequently, a total of 600 farmers were requested to provide seeds of their varieties grown last year, which ranged from one to maximum four varieties. After assessment of seed quality and germinability a total of 1,282 farmers’ seeds collected from 554 households were retained for subsequent analyses, so on average, the farmers cultivated two varieties in the aman season. In short, 100 g of rice seeds samples from up to four rice varieties grown by individual farmers were collected and stored in individual zipped-up plastic bags. All plastic bags were barcoded for Division, Distirct, Thana, Village, Household ID, and Variety ID at the collection site. A brief interview using computer-assisted personal interview (CAPI) software was conducted with the sample farmers, and information about the sample farmers and the names of all rice varieties that farmers cultivated were recorded.

Twenty-four breeder seeds from agricultural research centers in Bangladesh were selected and used as primary reference (Supplementary spreadsheet A) either because they were released aman varieties or released varieties reportedly being grown during aman. At the time of the initial survey in 2014 30 varieties from BRRI were listed as aman varieties (Supplementary spreadsheet A), of which 15 were available from BRRI upon request. Three aman varieties from BINA were available upon request at the time of the study (Supplementary spreadsheet A). Additionally, 6 BRRI varieties, released for boro or aus season were included, because they were popular among farmers during aman.

In 2016, the collected seed samples were visually assessed for purity using seed size, seed shape, hull color, pedicel color and awn morphology as indicators. Potential off-types were removed, and seeds were germinated in an experimental field in Bangabandhu Sheikh Mujibur Rahman Agricultural University (BSMRAU), Gazipur 1706. Leaf samples were collected and packed in plastic tubes with silica gel and were sent to the Genotyping Services Laboratory at IRRI headquarters in the Philippines, where DNA extraction was conducted based on the LGC sbeadex™ magnetic bead kit in combination with KingFisher Flex 96 from Thermo Scientific (www.fishersci.com)^25^.

### DNA fingerprinting, samples, and SNP quality control

DNA fingerprinting was performed using the Illumina Infinium 6K chip C6AIR^26^ genotyping platform, scoring 4,606 highly reliable SNP markers that are evenly distributed across the genome. Genotype call for each marker was inferred with GenomeStudio Software V2011.1 (Illumina, San Diego, CA). Quality control and data clean-up were performed to remove samples with a call rate lower than 0.80 (call rate <0.8) as well as samples with heterozygosity higher than 5% (He >0.05). SNP markers with minor allele frequency (MAF) ≤ 0.01 were also discarded from further analyses. To assess the uniqueness of each breeder seed, kinship coefficients between pairs of samples were estimated. Quality control analyses were performed using TASSEL version 3.0 software^40^.

### Genetic similarity estimate and cluster analysis

A genetic similarity matrix was computed using the simple matching coefficient of similarity implemented in the software Flapjack 1.16.10.31^41^ and genotypes were linked based on their amount of genetic similarity. Samples that did not match any of the breeder seeds used as a primary reference were compared to the accessions on the 3K SNP Seek (3K reference set)^23^ used as a secondary reference. The pattern of relatedness between breeder seeds was visualized by plotting the heat map of pairwise IBD estimate using the SNP & Variation Suite (SVS v8.6.0) (Golden Helix, Inc., Bozeman, MT, USA).

Cluster analysis was performed based on genetic distance between accessions using TASSEL version 3.0 software^40^. The genetic distance matrix generated by Tassel was used to create a phenogram based on the Unweighted Pair Group Method using Arithmetic Mean (UPGMA) algorithm^42^. The result phenogram was saved in the newick tree format and visualized using Figtree v.1.4.3 (http://tree.bio.ed.ac.uk/software/figtree/).

### Sub1 gene identification

Two Infinium 6K SNP markers were flanking the Sub1 gene on chromosome 9 (6.3–6.7 Mb): 9311693 (position 6.36 Mb) and 933955 (position 6.77 Mb). Of these, SNP 933955 can distinguish Sub1-introgressed varieties from their non-Sub1 counterparts. For example, from Swarna to Swarna_Sub1 and from BR11 to BR11_Sub1. All Sub1 varieties have the T allele whereas non-Sub1 varieties have the C allele. Thus, SNP 933955 was used to detect the presence of a tolerant allele of Sub1 in the MV. The suitability of this approach has been presented in detail in Thomson *et al*.^26^

### Bangladesh map generation

The map was downloaded from DIVA-GIS 7.5 (http://www.diva-gis.org) and modified using ggplot2^43^ in R^44^.

## Electronic supplementary material

Supplementary spreadsheet

Supplementary raw_data

## Data Availability

All data related to this study, including raw genotyping and survey data, is included as supplemental information (supplementary spreadsheets and supplementary raw_data) of this manuscript. It is further freely available upon request from IRRI (e.g.nmbanjo@irri.org and/or d.pagtananan@irri.org). Both IRRI and the Bill and Melinda Gates Foundation (BMGF), funder of this study, adhere to a strict open access policy. For further requests please contact the corresponding author via e-mail: t.kretschmar@scu.edu.au
